# Risk factors for suicide among South-East Asian women: A public health crisis in need of gender-specific solutions

**DOI:** 10.1371/journal.pmen.0000183

**Published:** 2024-11-14

**Authors:** Anil Fastenau, Priyanka Chahal, Amtul Shaheen, Mowmita Basak

**Affiliations:** 1 Marie Adelaide Leprosy Center, Karachi, Pakistan; 2 German Leprosy and Tuberculosis Relief Association (DAHW), Wuerzburg, Germany; 3 Department of Global Health, Institute of Public Health and Nursing Research, University of Bremen, Bremen, Germany; 4 Heidelberg Institute of Global Health, University of Heidelberg, Heidelberg, Germany; 5 Department of Global Health and Social Medicine, Harvard Medical School, Boston, Massachusetts, United States of America; 6 Department of Social and Cultural Anthropology, University of Cologne, Cologne, Germany; 7 Lepra, Colchester, United Kingdom; PLOS: Public Library of Science, UNITED KINGDOM OF GREAT BRITAIN AND NORTHERN IRELAND

Suicide represents a significant global public health issue, accounting for 70% of all fatalities among women caused by intentional harm and ranking as the third leading cause of death worldwide among females aged 15–29 [1]. Especially, in South-East Asia suicide is one of the leading causes of death among women, accounting for almost half of all female suicides worldwide. The South-East Asia Region (SEAR) has a much higher female age-standardized suicide rate (11.5 per 100 000) compared to the global female average (7.5 per 100 000) [2]. The WHO South-East Asia Region experiences a disproportionate number of suicides among young women, reflecting a troubling mix of socioeconomic, cultural, patriarchal and psychological factors [3]. While mental health is often the focus of global suicide prevention strategies, for South-East Asian women, gender-based stressors—such as domestic violence, forced marriage, and economic dependency—play a much more prominent role [4].

## Socioeconomic and demographic determinants

Research across South-East Asia consistently shows that young, married women from rural areas are especially vulnerable to suicide [5]. Women under 30, especially those with little education and no employment, are at the highest risk of attempting suicide [5]. Marriage, which is often seen as a protective factor for mental health globally, paradoxically increases suicide risk in the WHO SEAR [6]. Cultural expectations, such as the need to provide dowries or produce male children, combined with poverty and economic dependency, leave many women feeling trapped and helpless [7].

For women confined to ‘traditional’ housewife roles, the lack of financial independence is a significant contributor to suicidal behaviour. A study from Bangladesh shows that economic dependence on husbands or in-laws, coupled with the stress of marital conflicts and domestic abuse, can push women into situations where suicide seems like the only escape [8].

## Cultural and psychosocial stressors

Cultural norms and patriarchal structures in South-East Asia heavily influence female suicidality, particularly through entrenched gender disadvantage. In many parts of the region, practices like child marriage, forced marriage, and dowry disputes remain widespread, all of which contribute to the increased risk of suicide [9]. Child marriages often place young women in vulnerable situations, where they may face domestic violence, societal pressure, and a lack of personal autonomy.

Dowry disputes are another significant risk factor for suicide. In countries such as India and Bangladesh, dowry expectations from the in-laws place immense financial pressure on brides and their families [10]. Women who are unable to meet these demands often face severe abuse, leading many to resort to suicide as a way out [10]. The isolation and lack of support that many women experience, particularly in joint family systems, exacerbates these issues.

Gender-based violence, including intimate partner violence (IPV), is another major driver of suicidality in SEAR [11]. Women subjected to physical, emotional, or sexual abuse are much more likely to attempt or complete suicide [11]. The lack of external support systems and the frequent cultural acceptance of violence against women due to patriarchal structures in South-East Asia further compound the problem.

## Mental health and suicide

While mental health issues like depression and anxiety are commonly associated with suicide, they play a somewhat different role for South-East Asian women compared to women in high-income countries [12]. In South-East Asia, suicides are more likely to be triggered by external stressors—such as financial hardship, domestic abuse, or dowry disputes—rather than by mental illness alone [13]. Nevertheless, a study from India shows that untreated mental health conditions, particularly postpartum depression and anxiety, do contribute to suicidality [14].

The stigma surrounding mental health in this region prevents many women from seeking help [15]. Additionally, mental health services are limited, especially in rural areas where suicide rates are highest [16]. This lack of accessible and culturally appropriate mental health care along with reduced help-seeking behaviours, exacerbate the challenges faced by vulnerable South-East Asian women.

## Recommendations for suicide prevention for South-East Asian women

To effectively address the crisis of female suicide in the WHOSEAR, comprehensive and culturally sensitive strategies are needed that tackle both the psychological and sociocultural factors contributing to suicidality. Below are key recommendations:

## 1. Implement gender-specific suicide prevention strategies

Suicide prevention efforts must be tailored to the specific needs of women in this region. Policies should focus on addressing the root causes of female suicidality, such as domestic violence, child marriage, and dowry disputes. Governments should prioritize legal frameworks that protect women from gender-based violence and ensure that perpetrators are held accountable.

National suicide prevention strategies should incorporate a gender-sensitive lens, recognizing that women’s experiences of suicide are often distinct from men’s. Mental health services, legal protections, and support networks must be designed to address the unique challenges faced by women in South-East Asia.

## 2. Strengthen legal protections and gender equality

Legal reforms aimed at protecting women from domestic violence, forced marriage, and dowry disputes are essential to reducing female suicidality. Governments in SEAR must ensure that women have access to legal recourse and that domestic abusers and those involved in dowry-related violence are prosecuted. Legal protections should also allow women to leave abusive marriages without fear of financial ruin or social stigma.

Promoting gender equality should be central to any suicide prevention strategy in the region. Expanding access to education and employment opportunities for women can provide them with the financial independence needed to escape abusive relationships. Empowering women economically is crucial for reducing their vulnerability to suicide and improving their well-being.

## 3. Improve mental health services and accessibility

Mental health services in South-East Asia are often under-resourced and inaccessible, particularly in rural areas. Governments must invest in mental health infrastructure to ensure that women have access to affordable and culturally sensitive care. This includes training health providers to recognize the common mental health challenges faced by women in this region, such as postpartum depression.

Efforts to reduce the stigma surrounding mental health treatment are also vital. Public health campaigns should raise awareness of mental health issues and encourage women to seek help when needed. Community-based mental health services, especially in rural areas, should be expanded to reach vulnerable populations such as young, married women.

## 4. Address rural vulnerability

Most suicides among women in South-East Asia occur in rural areas, where access to education, employment, and health services is limited. Suicide prevention strategies must address the unique vulnerabilities faced by rural women, including expanding economic opportunities and improving social support networks in remote areas.

Programs that restrict access to lethal means, such as pesticides—which are often used in rural suicides—should be a top priority [17]. Increasing women’s access to livelihood options and improving their economic resilience can help reduce the financial pressures that contribute to suicidality.

## 5. Promote education and empowerment

Education can play a key role in mitigating factors that contribute to suicide in SEAR. Women with higher levels of education in this region are less likely to experience early marriage, economic dependence, or domestic violence—key risk factors for suicide. Local governments should prioritize education for girls and women, especially in rural areas where dropout rates are higher.

Empowerment programs that enhance women’s self-esteem and autonomy are crucial in reducing suicidality in South-East Asia. These programs should be integrated into community health and education systems to equip women with the tools they need to navigate crises without resorting to suicide.

## Conclusions

Female suicide in South-East Asia is a complex public health crisis driven by gender disadvantage, domestic violence, poverty, patriarchal norms and cultural expectations. Comprehensive, gender-specific suicide prevention strategies are urgently needed to address the unique challenges that women in this region face. By strengthening legal protections, expanding mental health services, and promoting education and economic empowerment, South-East Asian countries can take significant steps toward reducing female suicidality. Immediate action is required to prevent more women from losing their lives to a crisis that is largely preventable with the right interventions.
